# The Effect of Thigh Muscle Forces on Knee Contact Force in Female Patients with Severe Knee Osteoarthritis

**DOI:** 10.3390/bioengineering11121299

**Published:** 2024-12-20

**Authors:** Tingting Liu, Hao Xie, Songhua Yan, Jizhou Zeng, Kuan Zhang

**Affiliations:** 1 School of Biomedical Engineering, Capital Medical University, Beijing 100071, China; ltt@ccmu.edu.cn (T.L.); 122019000109@ccmu.edu.cn (H.X.); yansh74@ccmu.edu.cn (S.Y.); 2 Department of Orthopedics, Beijing Luhe Hospital, Capital Medical University, Beijing 101100, China

**Keywords:** musculoskeletal model, knee osteoarthritis, muscle force, knee contact force, maximum isometric force, ultrasound

## Abstract

Thigh muscles greatly influence knee joint loading, and abnormal loading significantly contributes to the progression of knee osteoarthritis (KOA). Muscle weakness in KOA patients is common, but the specific contribution of each thigh muscle to joint loading is unclear. The gait data from 10 severe female KOA patients and 10 controls were collected, and the maximum isometric forces of the biceps femoris long head (BFL), semitendinosus (ST), rectus femoris (RF), vastus lateralis (VL), and vastus medialis (VM) were calibrated via ultrasound. Four musculoskeletal (MSK) models were developed based on EMG-assisted optimization, static optimization, and ultrasound data. The ultrasound-calibrated EMG-assisted MSK model achieved higher accuracy (R^2^ > 0.97, RMSE < 0.045 Nm/kg). Patients exhibited increased VL and VM forces (*p* < 0.004) and decreased RF force (*p* < 0.006), along with elevated medial and total joint contact forces (*p* < 0.001) and reduced lateral forces (*p* < 0.001) compared to controls. The affected side relied on VL and BFL the most (*p* < 0.042), while RF was key for the unaffected side (*p* < 0.003). Ultrasound calibration and EMG-assisted optimization significantly enhanced MSK model accuracy. Patients exerted greater quadriceps and hamstring forces bilaterally, shifting knee loading medially, and depended more on the lateral thigh muscles on the affected side. Hamstrings contributed more to joint contact forces, while quadriceps’ contributions decreased.

## 1. Introduction

Knee Osteoarthritis (KOA) is a prevalent chronic degenerative disease that has become one of the leading causes of disability worldwide [1]. The global prevalence of KOA is as high as 22.9% in individuals aged 40 and over [2]. As age advances, the instability of joints and cartilage damage exacerbates, increasing the risk of KOA, which peaks around the age of 50 [3]. Up to 60% of individuals suffering from KOA are female [4], exhibiting a 2.6-fold prevalence rate compared to men [5]. The female gender also constitutes a significant risk factor for the progression of the disease [6]. In China, from 2011 to 2020, more than three-quarters of patients undergoing joint replacement surgery for severe KOA were women [7]. These epidemiological data indicate that more research should focus on the biomechanics of KOA in women. Abnormal knee joint loading and its distribution are key factors contributing to the progression of KOA [8]. The muscles surrounding the joint are crucial for stabilizing the knee during daily activities, serving as the primary source of joint loading and as the main regulators of its distribution [9,10]. KOA is often associated with a marked decline in thigh muscle function, including muscle weakness and abnormal muscle activation [11,12]. The degree of muscle dysfunction intensifies with the progression of the disease [13], which undoubtedly alters the muscles’ effect on joint loading and distribution [14,15]. However, no studies have investigated the relative contributions of individual muscles to knee joint contact loading during walking in female patients with severe KOA.

To accurately analyze the contributions of individual muscles to joint loading in KOA patients, precise calculation of muscle forces is essential. Musculoskeletal (MSK) modeling, an emerging technique for gait analysis and muscle force estimation, has been widely applied to the KOA population [16,17]. Yet, accurate computation of thigh muscle forces during walking in KOA patients remains challenging. This is primarily due to specific alterations in thigh muscle function among KOA patients. Firstly, muscle atrophy and weakness are common in female patients with KOA, with muscle degeneration varying in degree and leading to marked individual differences between actual muscle parameters and the default parameters used in MSK models [18,19]. Muscle parameters, such as the maximum isometric force, tendon slack length, and optimal muscle length [20], are particularly critical to the predictive performance of models. Among these, the maximum isometric force has a significant impact on the model’s predictive capabilities [21]. Calibrating muscle parameters like tendon slack length and optimal muscle length alone marginally improves joint torque prediction accuracy, whereas adjusting maximum isometric force significantly boosts precision. Numerous studies have made efforts to calibrate the maximum isometric force. In recent years, the Calibrated EMG-informed NMS Modelling Toolbox (CEINMS) has comprehensively enabled the calibration of muscle parameters, including maximum isometric force, tendon slack length, and optimal muscle length [22]. However, its approach of using a uniform scaling factor to adjust the maximum isometric force does not provide an effective solution for the muscle weakness issue in KOA patients [23,24]. In KOA patients, the degree of muscle degeneration varies among the compartments of the thigh muscles [13], with the changes in maximum isometric force differing across muscles. This makes the use of a uniform scaling factor for reduction an unrealistic approach to simulate muscle weakness, as it does not align with the actual conditions of the subjects [25]. Charles et al. [26] personalized muscle parameters using MRI in young individuals, significantly improving knee joint torque prediction accuracy compared to the general model based on elderly cadaver data (13.2% vs. 57.3% root mean square error). Oliveira et al. [27] minimized ankle joint torque calculation errors by calibrating maximum isometric muscle forces in young adults with ultrasound data, achieving up to 14% error reduction. Combining magnetic resonance imaging (MRI) and ultrasound techniques can enhance the accuracy of these calculations [23,28]. but the complexity, high cost, and time-consuming nature of data collection limit their widespread application. With advancements in ultrasound technology for accurate muscle volume measurement, the muscle’s maximum isometric force can be determined by using the ultrasound alone [29]. Secondly, alterations in muscle function among KOA patients are also evident in abnormal muscle activation patterns, characterized by high levels of muscle co-activation and significant inter-individual variability [30]. The MSK system exhibits significant redundancy, allowing for a multitude of muscle force configurations that yield identical joint kinematics and kinetics [31]. Solutions to address this redundancy are achieved through computational algorithms, which include static optimization and the electromyography (EMG)-assisted approach [32]. Static optimization algorithms synthesize all muscle excitations without the use of experimental EMG data [22]. It may not capture the unique muscle activation patterns and/or co-contraction strategies frequently observed in the KOA patient population, leading to significant inaccuracies in KOA patients’ muscle force calculations [33]. EMG-assisted algorithms adjust excitations based on experimental EMG signals and synthesize excitations for muscles without available experimental EMG data [22]. EMG-assisted algorithms offer an advantage in simulating abnormal muscle activation and hold promise for providing more accurate solutions to muscle force estimation in KOA patients [34]. The EMG-assisted method combining in vivo imaging data for muscle parameter calibration has not been applied to gait analysis in severe KOA female patients.

Accurate computation of muscle forces during walking and the analysis of the individual muscle contributions to knee joint loading are crucial for developing targeted rehabilitation strategies for KOA patients and slowing the progression of the disease. The objectives of the research include: (1) constructing an ultrasound-calibrated EMG-assisted MSK model for the accurate estimation of muscle forces; (2) comparing the differences in thigh muscle forces and joint contact forces during gait between KOA patients and healthy controls; and (3) exploring the differential contribution patterns of thigh muscle forces to joint contact forces between patients and healthy controls.

## 2. Materials and Methods

### 2.1. Subjects

Ten healthy elderly female participants who had no history of musculoskeletal or neurological disorders and ten female patients with severe KOA were recruited. The patient inclusion criteria were as follows: (1) meeting the diagnostic criteria for KOA established by the American College of Rheumatology [35]; (2) having a Kellgren–Lawrence (KL) grade of III–IV [36]; and (3) being able to walk independently. The exclusion criteria [37] included: (1) having neurological disorders such as stroke or Parkinson’s disease; (2) suffering from muscle diseases such as myasthenia gravis, progressive muscular dystrophy, or periodic paralysis; (3) having a history of significant trauma or surgery; and (4) having other inflammatory arthritis. There were no statistically significant differences in age, height, weight, or body mass index (BMI) between the control group and the KOA group (Table 1). The study was approved by the Ethics Committee of Capital Medical University and adhered strictly to the ethical principles outlined in the Declaration of Helsinki. All participants provided written informed consent after a thorough understanding of the study procedures.

### 2.2. Data Collection

A six-camera Motion Analysis motion capture system (Motion Analysis, Rohnert Park, CA, USA) was used to acquire marker trajectory data from participants at a sampling frequency of 120 Hz. Concurrently, a Kistler three-dimensional force measurement platform (Kistler, Amherst, NY, USA) was employed to collect ground reaction force data during walking at a sampling rate of 1000 Hz. The Delsys Trigno wireless electromyography system (Delsys Trigno wireless system; Natick, MA, USA) was employed to concurrently capture the electromyographic activity from five key muscles in each thigh: the long head of the biceps femoris long head (BFL), semitendinosus (ST), rectus femoris (RF), vastus lateralis (VL), and vastus medialis (VM), at a sampling rate of 2000 Hz while the participants were walking. The placement of surface EMG electrodes adhered to the SENIAM guidelines [38]. Participants walked barefoot on a 10-m-long walkway at their normal walking speed, with each leg being tested for 10 valid trials. For both marker trajectory and ground reaction force data, a fourth-order 6 Hz Butterworth zero-lag filter was applied for low-pass filtering. The EMG signals were initially processed with band-pass filtering, full-wave rectification, and low-pass filtering, followed by normalization based on the maximum EMG amplitude during the movement.

An Aixplorer real-time shear wave elastography ultrasound system (Aixplorer; SuperSonic Imagine, Aix-en-Provence, France) with an L10-2 linear array probe, combined with wide-field imaging technology, was used to perform ultrasound measurements of the BFL, ST, RF, VL, VM muscles in the participants. During the measurement, participants were required to lie in a supine or prone position and remain fully relaxed. Anatomical landmarks around the knee joint were used as reference points to ensure consistent data collection locations across all participants [39] (Table 2). Each measurement was repeated three times, with a minimum interval of 3 s between each ultrasound image capture. All ultrasound images were collected by a physician with five years of experience in skeletal muscle ultrasound imaging. The ImageJ software (version 1.2.4) (National Institutes of Health, Bethesda, MD, USA) was used to process the ultrasound images and obtain muscle thickness, pennation angle, and cross-sectional area [40]. Muscle volumes were calculated using regression equations for each muscle’s cross-sectional area (CSA) and volume from previous studies [41,42]. While the participant was standing relaxed, the thigh length (distance from the greater trochanter of the femur to the joint space) was measured. The distal end of the thigh, located at the knee joint space, was designated as the 0% mark. For healthy participants, the CSA measurement for the quadriceps was taken at 50% of the thigh length, and for the hamstrings, it was taken at 40% from the distal end of the thigh. For KOA patients, the CSA measurements for both the quadriceps and hamstrings were taken at 40% from the distal end of the thigh. The physiological cross-sectional area (PCSA) of the muscles was calculated using the formula [28] (Table 3). Following the methodology of Menegaldo et al. [21] the maximum isometric force of the muscles in the musculoskeletal model was calculated based on the participants’ PCSA (Table 4).

(1) $${MV}_{H}=FL\times (CSA\times Intercept+Slope)$$

(2) $${MV}_{P}=Intercept\times FL\times CSA+Slope$$

(3) $$L_{f}=MT/sin\alpha$$

(4) $$PCSA=MV\times \cos\alpha /L_{f}$$

(5) $$F_{o}^{M}=PCSA\times \sigma$$

${MV}_{H},\;{MV}_{P}$ represent the muscle volumes of healthy individuals and patients, respectively; FL denotes the thigh length; MV indicates the volume of an individual muscle, α represents the pennation angle, $L_{f}$ is the muscle fiber length, PCSA represents the maximum isometric force, σ denotes the maximum specific muscle force (61$N/{cm}^{2}$ [43]).

**Table 2:** Measurement site.

Muscle	Measurement Site
RF	Lower one-third of the line connecting the anterior superior iliac spine and the upper edge of the patella
VL	Lower fifth of the line connecting the greater trochanter of the femur and the medial condyle of the femur
VM	Lower one-third of the line connecting the greater trochanter of the femur and the lateral condyle of the femur
BFL	The lower third of the line connecting the ischial tuberosity and the lateral popliteal fossa
ST	The lower third of the line connecting the ischial tuberosity and the medial popliteal fossa

RF, Rectus Femoris; VM, Vastus Medialis; VL, Vastus Lateralis; BFL, Biceps Femoris long head; ST, Semitendinosus.

**Table 3:** The intercept and slope of the muscle volume prediction equation.

Muscle	Healthy Group		KOA Group	
	Intercept	Slope	Intercept	Slope
RF	0.749	0.717	0.56	17.9
VL	0.581	0.529	0.54	8.1
VM	1.329	0.556	0.44	32.3
BFL	0.211	0.326	0.31	5.0
ST	0.687	0.431	0.41	18.2

**Table 4:** Estimated maximum isometric force.

	KOA Group		Healthy Group	95% CI	*p*-Value
	Symptomatic Side	Asymptomatic Side			
RF	979.6 (803.2~1048.8)	1024.6 (968.0~1082.7)	1135.5 (1046.1~1179.2)	196.5 (147.9~256.1)	<0.001 **
VL	1725.7 (1592.3~1820.2)	1898.1 (1724.5~2063.6)	2163.1 (2089.6~2296.3)	484.5 (422.5~542.6)	<0.001 **
VM	1330.8 (1231.7~1425.8)	1307.4 (1208.9~1450.9)	1397.8 (1327.0~1490.2)	132.2 (78.2~187.7)	0.035 *
BFL	720.3 (671.2~790.4)	771.3 (736.4~843.5)	885.3 (799.2~925.9)	115.5 (78.2~154.8)	<0.001 **
ST	401.0 (374.4~427.1)	410.3 (398.7~446.4)	425.6 (390.2~508.1)	37.4 (19.5~57.5)	0.017 *

* *p* < 0.05 ** *p* < 0.01.

### 2.3. Model Development

**Figure 1:**
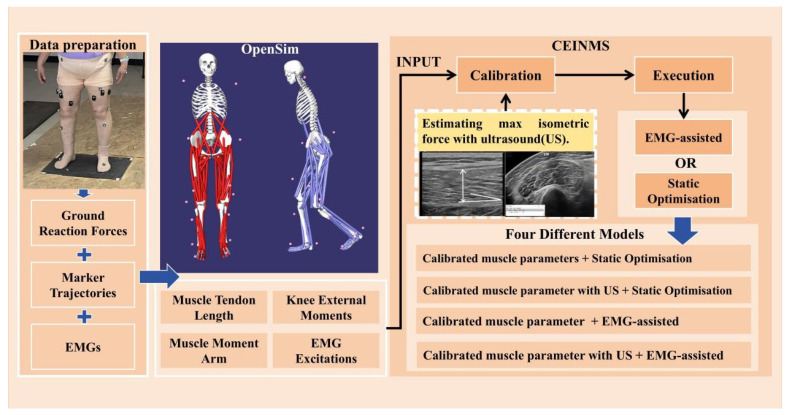
MATLAB was utilized for the initial processing of experimental data, encompassing marker trajectories, ground reaction forces (GRFs), and electromyography (EMG) signals. This processing involved filtering, normalizing EMG signals, and converting the data for compatibility with OpenSim. Utilizing OpenSim’s APIs, we scaled the model and performed inverse kinematic analysis, muscle analysis, and inverse dynamics analysis to ascertain muscle–tendon unit (MTU) lengths, moment arms, and joint moments. Muscle parameters were anthropometrically adjusted and integrated into the CEINMS calibration procedure to enhance parameter accuracy, thereby reducing the difference between experimental and predicted joint moments. Ultrasound data was employed to calibrate the maximum isometric force. By integrating two distinct neural control algorithms with varying levels of muscle parameter calibration, we ultimately developed four unique models. The CEINMS execution module utilized the refined muscle activation data, in conjunction with MTU lengths and moment arms from OpenSim, to estimate MTU forces and joint moments from individual experimental trials.

Gait biomechanical analysis was conducted using the OpenSim generic model (gait2392, version 3.2) (Figure 1), and the calculation of the medial and lateral knee joint contact forces was achieved by adding degrees of freedom and contact points to the knee joint [10]. Based on the spatial information of markers collected during static standing, the generic model was linearly scaled, and the parameters of some muscle–tendon units (MTUs), muscle attachment points, model centroid coordinates, and moments of inertia were scaled by the same proportion. Subsequently, the Inverse Kinematics (IK) and Inverse Dynamics (ID) tools were employed to calculate the knee joint kinematics and the knee joint moments. Using the Muscle Analysis tool, we obtained the time-varying muscle length data and the moment arms of muscles with respect to the knee joint moments. In conjunction with the CEINMS, the relevant muscle parameters were calibrated. The linearly scaled tendon slack length and optimal fiber length were used as inputs in the calibration process. During calibration, the tendon slack length and optimal fiber length for each MTU were constrained to within 75% of their initial values. The activation dynamics parameters A, C1, and C2 were globally calibrated, with the shape factor A ranging from −3 to 0 and the coefficients C1 and C2 ranging from −1 to 1. The range for the intensity factor was set between 0.5 and 2.5. On the basis of the preliminary calibration and without altering the intensity factor, further calibration of the muscle parameters was conducted using the maximum isometric force derived from ultrasound measurements [23]. This study employed both static optimization and EMG-assisted optimization neural control algorithms to calculate the muscle forces during walking. In both cases, the following objective function was solved frame by frame [22]:

(6) $$f=\alpha E_{Moment}+\beta E_{sumEXC}+\gamma E_{EMG}$$

$E_{sumEXC}$ represents the sum of the squares of muscle activations; $E_{Moment}$ and $E_{EMG}$ denote the errors between the experimental and estimated joint moments and muscle activations, respectively; α, β, and γ are the weighting factors adjusted according to different neural solution configurations.

For the static optimization, parameters α and β are set to 1 and 2, respectively, while γ is set to 0. For the EMG-assisted method, parameter α is set to 1, and parameters β and γ are adjusted as needed to minimize error, thereby better tracking the linear envelope of the EMG signals and the experimental joint moments. We ultimately constructed four models: (1) a musculoskeletal model incorporating the static optimization method based on muscle parameter calibration (SO); (2) a musculoskeletal model using the static optimization method with muscle parameter calibration based on the estimated maximum isometric force from ultrasound measurements (SO_US); (3) a musculoskeletal model incorporating the EMG-assisted optimization method based on muscle parameter calibration (EMGAss); (4) a musculoskeletal model using the EMG-assisted optimization method with muscle parameter calibration based on the estimated maximum isometric force from ultrasound measurements (EMGAss_US).

### 2.4. Data Analysis

This study utilized the coefficient of determination ($R^{2}$) to assess the accuracy of different models in calculating joint moments and muscle activations. The root mean square error (RMSE) was used to quantify the discrepancy between the model calculations and the inputs. The most accurate model was used to further calculate the muscle forces and joint contact forces during the stance phase for patients and healthy individuals. The study compared the average muscle forces and joint contact forces at various percentage time points during the stance phase between patients and healthy individuals, as well as the peak muscle forces and joint contact forces. The Shapiro–Wilk test confirmed the normality of data across variables. Statistical analysis was conducted using *t*-tests if the data met the normal distribution criteria; otherwise, the Wilcoxon signed-rank test for paired samples was employed. Linear regression analysis was used to investigate the contribution of individual thigh muscle force to knee joint contact forces in both patients and healthy controls. All statistical analyses were performed using SPSS 26.0 software, with a significance level set at α = 0.05.

## 3. Results

### 3.1. Model Accuracy in Knee Joint Moments

For both the patients and healthy controls, the EMGAss_US model exhibited the highest level of accuracy in calculating knee joint moments, with R^2^ greater than 0.97 and RMSE less than 0.045 Nm/kg (Table 5). In contrast, the SO model demonstrated a relatively inferior performance in replicating joint moments, with R^2^ value less than 0.874 and RMSE exceeding 0.086 Nm/kg. The calibration of the maximum isometric force using ultrasound data significantly enhanced the precision of knee joint moment calculations (*p* < 0.001).

### 3.2. Model Accuracy in Muscle Excitations

For each muscle across all study populations, the muscle excitations calculated by the EMG-assisted model were closer to experimental muscle excitations compared to the static optimization model, with higher R^2^ and substantially lower RMSE (*p* < 0.001) (Table 6). The calibration of the maximum isometric force using ultrasound data did not enhance the accuracy of muscle activation calculations for either the patient or the healthy control group (*p* > 0.05).

### 3.3. Muscle Force

In average muscle forces, the affected side of patients showed higher forces in VL and VM compared to healthy controls (*p* < 0.001), with lower forces in RF and ST (*p* < 0.001) (Table 7). The contralateral side had increased forces in ST, VL, and VM (*p* < 0.001) but reduced forces in BFL and RF (*p* < 0.001). Compared to the contralateral side, the affected side had greater forces in RF, VL, VM, and BFL (*p* < 0.001). For peak muscle forces, the affected side had higher peaks in VL and VM (*p* < 0.004) but lower RF peaks on both sides versus controls (*p* = 0.006). The ST peak force was also lower on the affected side than on the contralateral side (*p* = 0.003).

### 3.4. Knee Contact Force

**Figure 2:**
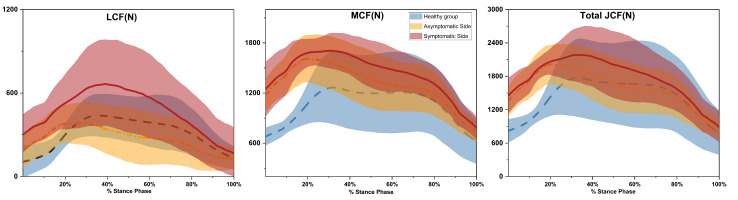
Comparisons of knee contact force disparity.

Both the medial and total average joint contact forces were higher in patients than in the healthy control group (*p* < 0.001) (Table 8). The affected side saw increases of 39% and 24% for the medial and total forces, respectively, while the contralateral side increased by 35% and 17%. Lateral joint contact forces were lower in both patient sides compared to controls (*p* < 0.001), with decreases of 32% and 42%. The affected side’s lateral force was 17% higher than the contralateral side (*p* = 0.003). For peak joint contact forces, the first peak of the medial and total forces on the affected side was greater in patients than in controls (*p* < 0.018) (Figure 2). The first and second peaks of the lateral forces were lower (*p* < 0.007). The first peak of the total force on the contralateral side was also greater in patients (*p* = 0.038).

### 3.5. Muscle Contributions to Knee Contact Force

**Figure 3:**
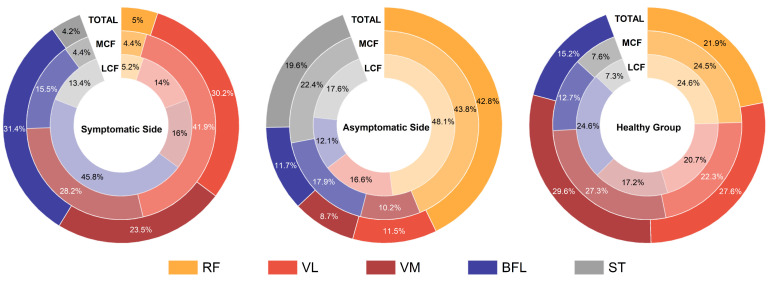
Muscle contributions to knee contact forces during the stance phase.

On the affected side, the primary contributors to the joint contact force were the VL, VM, and BFL, with contribution rates reaching 43.3%, 29.8%, and 48.5%, respectively (*p* < 0.042) (Figure 3). On the unaffected side, the RF made the most significant contribution to the joint contact force, accounting for 50.9% of the total (*p* < 0.003). In contrast, the contributions of the muscles to joint contact force in the healthy control group were more evenly distributed, ranging from 13.4% to 28.8% (*p* < 0.003).

## 4. Discussion

Four different musculoskeletal models were developed based on EMG optimization, static optimization, and ultrasound data, and the results show that the model calibrated with ultrasound data for maximum isometric force and utilizing EMG-assisted optimization performed the best in KOA patients and healthy controls. Compared to a healthy control group, KOA patients exhibited a significant overall increase in the muscle forces of both the quadriceps and hamstring muscles. The fluctuations in bilateral joint contact forces in KOA patients were more subdued, with less pronounced peaks. The medial and total joint contact forces were significantly higher in KOA patients compared to healthy controls, whereas the lateral joint contact forces were significantly lower. Among KOA patients, the vastus lateralis and biceps femoris long head on the affected side made a substantially greater contribution to joint contact forces, while the contralateral side exhibited a greater reliance on the rectus femoris. Additionally, the relative contribution of the hamstrings to joint contact forces increased, whereas that of the quadriceps decreased.

Mean knee contact force during the stance phase is depicted for three groups: healthy individuals (blue), the asymptomatic side of KOA patients (yellow), and the symptomatic side of KOA patients (red). Data are presented as mean values with standard deviations (SD). The dashed line indicates the healthy group, the dash-dot line represents the asymptomatic side, and the solid line denotes the symptomatic side.

The EMG-assisted optimization algorithm demonstrates superior performance over the static optimization algorithm in both calculating knee joint moments and estimating muscle excitation accuracy. The poor performance of the static optimization algorithm may be due to its consideration of activation dynamics within the optimization loop, where muscle activation is influenced by historical neural excitation values [22]. Previous studies have indicated that the static optimization algorithm may not adequately capture abnormal muscle activation patterns or co-contraction strategies in populations with neuromuscular dysfunction [34]. Our findings support this hypothesis. KOA patients often exhibit neuromuscular alterations [11] characterized by elevated levels of muscle activation and increased co-contraction of lateral knee muscles. Compared to the static optimization algorithm, the EMG-assisted optimization algorithm significantly improves the accuracy of muscle excitation calculations in KOA patients, providing a more precise reflection of their abnormal muscle activations. Davico et al. [23] also found that EMG-assisted optimization algorithms can more effectively capture pathologically related muscle activation abnormalities. In children with cerebral palsy, the estimated muscle excitation using a static optimization model was significantly inferior compared to the EMG-assisted model (R^2^ = 0.221 versus R^2^ = 0.623). Additionally, calibrating the model with ultrasound data for maximum isometric muscle force further enhances the precision of joint moment calculations, offering a more accurate simulation of the muscle atrophy and weakness prevalent in KOA patients [18]. In summary, the construction of musculoskeletal models benefits significantly from the use of in vivo medical imaging to accurately represent the subjects’ physiological conditions, thereby enhancing the computational accuracy of the models.

Compared to the healthy control group, KOA patients demonstrated elevated overall muscle forces in both the quadriceps and hamstrings on the affected side. Significantly increased average muscle forces were noted in the VL and VM muscles, with the peak muscle forces of the VL, VM, BFL, and ST muscles nearly doubling those of the controls. Ghazwan et al. [24] utilized an EMG-driven musculoskeletal knee model and found that, compared to healthy controls, patients with unicondylar KOA exhibited increased muscle force in the quadriceps and hamstring during the stance phase, with a 25% increase in peak force observed in the hamstrings. The increased overall muscle force of the quadriceps and hamstrings on the affected side may serve to support the joint and counteract excessive knee adduction moments [44]. The quadriceps, playing a pivotal role in knee joint control, are immediately activated post-heel strike to prevent excessive or rapid knee flexion [45]. As antagonists to the quadriceps, the hamstrings are known to exhibit co-activation in KOA patients [46,47]. While the co-contraction of the quadriceps and hamstrings may lead to increased knee stiffness, this could be a common strategy adopted by patients with weakened thigh muscles to maintain gait stability [48]. It is noteworthy that our findings reveal a decrease in the bilateral RF muscle force in patients with KOA compared to healthy individuals. Amiri et al. [49] employed an EMG-driven musculoskeletal model to estimate muscle force, and they found that patients with moderate medial osteoarthritis who exhibited radiographic progression of KOA had lower RF muscle force compared to those without radiographic progression of KOA. In this study, among all the muscles examined, the RF in female patients with severe knee osteoarthritis exhibited a significant degree of atrophy in maximum isometric force (a reduction of 13.7%), which is speculated to be one of the factors contributing to their impaired muscle force during walking. Knee joint pain and the inhibitory effects of joint afferent information on neural activation significantly restrict the normal function of the thigh muscles in KOA patients [50], which is likely also adversely affecting the RF muscle.

Relative contributions of individual muscle forces to the lateral (LCF), medial (MCF), and total (Total JCF) joint contact forces are presented from left to right for the symptomatic side, asymptomatic side, and healthy group. Muscle abbreviations are as follows: RF—Rectus Femoris, VM—Vastus Medialis, VL—Vastus Lateralis, BF—Biceps Femoris long head, ST—Semitendinosus.

We found that the medial and total joint contact forces on both sides of KOA patients were significantly higher than those in the healthy control group, with increases of 35% and 24%, respectively. In contrast, lateral joint contact forces were reduced by 48% relative to the controls, indicating that KOA patients shifted the load within the knee joint from the lateral compartment to the medial compartment. Unlike the distinct double peaks observed in the joint contact force curves of healthy individuals, the curves of KOA patients exhibited a smoother profile with less pronounced peaks. On the affected side, the first peak of the medial and total joint contact forces was 1.18 and 1.19 times higher than those of healthy individuals, respectively, while the first and second peaks of the lateral joint contact forces were reduced by 32% and 40%. Dell’Isola et al. [51] found that the peak medial joint contact force in KOA patients with varus deformity was 1.08 times that of healthy individuals, while the peak lateral joint contact force was reduced by 31%. Compared to healthy individuals, Zhang et al. [17] found that the peak medial and total joint contact forces in KOA patients with KL grades 3–4 increased by 83.6% and 49.4%, respectively. There were no significant differences in joint contact forces between the affected and non-affected sides in KOA patients, which may be because the patients have severe KOA. As the degenerative changes in the knee joint progress, the asymmetry of loading on both knees in KOA patients tends to decrease [52]. Moderate KOA patients might alleviate pain on the affected side during movement by transferring weight to the contralateral side, thereby increasing contralateral joint contact forces, but this phenomenon is not observed in patients with more severe deformities [53].

Significant differences in the contributions of various muscles to joint contact forces were observed between KOA patients and healthy individuals. In both the patient group and the healthy controls, the collective contribution of the quadriceps muscles was greater than that of the hamstrings. On both sides of KOA patients, there was an increase in the relative contribution of the hamstrings to joint contact forces, concurrently with a decrease in the relative contribution of the quadriceps. The affected side’s hamstrings contributed 37.7% to the total joint contact force, significantly higher than the 16.1% in healthy individuals, while the quadriceps’ contribution was 62.3%, much lower than the 83.9% in the healthy group. The contribution of individual muscles to joint contact forces in patients is significantly elevated. Notably, the VL and BFL on the affected side made the most significant contributions to joint contact forces, reaching as high as 43.3% and 48.5%, respectively. The significant increase in the contribution of the VL and BFL on the affected side is not unexpected. As the disease progresses, KOA patients experience an increase in the adduction moment, which leads to enhanced muscle activation and a further increase in the co-contraction of the lateral muscles around the knee joints [30,45,54]. The unaffected side relies more heavily on the RF, which contributes 50.9% to joint contact forces, potentially serving as a mechanism to preserve balance and control during locomotion. To alleviate pain and ensure stability, individuals with KOA frequently adjust their load distribution, increasing the load on the healthier side to diminish the strain on the affected knee joint [53]. As the only muscle in the quadriceps that crosses the knee joint, the RF is instrumental in the load reallocation process and has the unique capacity to compress not only a single compartment but both the medial and lateral compartments [55]. Consequently, the unaffected side exhibits a greater reliance on the augmented force of the RF to achieve a more comprehensive alteration of the joint load.

This study has certain limitations. The small sample size may have constrained the generalizability and universality of the results. Although our study demonstrated a high level of statistical power, the findings might not be representative of a broader population. Future research should aim to increase the sample size to enhance the widespread applicability of the conclusions. Only female patients with severe KOA were included in the present study, which may limit the generalizability of our findings. However, given the much higher prevalence of KOA in women, the findings still hold significant clinical importance. Only female patients with severe KOA (KL grades III & IV) were included in the present study, which may limit our understanding of the dynamic changes in muscle function throughout the disease progression. Future longitudinal studies should examine how muscle function evolves across different KL grades and its relationship with knee contact forces. Beyond alterations in thigh muscle functions, other potential factors such as structural changes within the joint, disease severity, obesity, and physical activity levels all influence knee joint loading and should be considered in future studies. Our study compared the muscle forces and joint contact forces during the stance phase between patients and healthy controls using average and peak forces. Future research could enhance the analysis by examining the forces at various stages of the gait cycle, which may offer a deeper understanding of the variability of biomechanics in KOA patients.

## 5. Conclusions

Ultrasound calibration and EMG-assisted optimization significantly enhance the accuracy of MSK models for the prediction of muscle forces. During walking, KOA patients demonstrate a notable increase in the overall muscle forces of both the quadriceps and hamstrings, along with an increase in medial and total joint contact forces and a reduction in lateral joint contact forces. On the affected side, the vastus lateralis and biceps femoris long head have a greater influence on joint contact forces, with an increased reliance on the lateral thigh muscles and the hamstrings. The unaffected side relies more on the rectus femoris. These findings highlight the critical role of muscles in joint load distribution and may help to develop personalized and targeted rehabilitation strategies to slow the progression of KOA.

## Figures and Tables

**Table 1:** Participant characteristics.

	KOA Group (n = 10)	Healthy Group (n = 10)	*p*-Value
Age (y)	65.5 (63.8~69.3)	68.0 (66.8~69.0)	0.303
Height (cm)	158.0 (154.8~160.8)	157.0 (155.8~164.5)	0.761
Weight (kg)	67.5 (61.8~71.5)	65.5 (63.5~68.8)	0.622
BMI (kg/m^2^)	26.3 (25.3~29.0)	26.2 (24.1~26.9)	0.384
KL grade	III/IV (2/8)	—	—
Walking Speed (m/s)	0.96 (0.80~1.10)	1.06 (0.9~1.1)	0.185

Values are presented as median (interquartile range). KOA, Knee osteoarthritis; BMI, Body mass index; KL, Kellgren–Lawrence.

**Table 5:** Model accuracy in knee joint moments.

		EMGAss	EMGAss_US	SO	SO_US	Effect Size	*p*-Value
Symptomatic Side	R^2^	0.928 (0.824, 0.993)	0.985 (0.934, 0.998)	0.812 (0.557, 0.936)	0.925 (0.819, 0.970)	0.36	*p* < 0.001
	RMSE (NM/kg)	0.054 (0.028~0.088)	0.027 (0.008~0.050)	0.1 (0.055~0.140)	0.055 (0.04~0.095)	0.339	*p* < 0.001
Asymptomatic Side	R2	0.919 (0.859, 0.995)	0.970 (0.890, 0.997)	0.874 (0.722, 0.919)	0.947 (0.827, 0.990)	0.1809	0.001 ***
	RMSE (NM/kg)	0.071 (0.025~0.088)	0.045 (0.028~0.075)	0.086 (0.075~0.103)	0.062 (0.023~0.073)	0.1794	0.001 ***
Healthy Group	R^2^	0.973 (0.879, 0.996)	0.990 (0.974, 0.996)	0.753 (0.481, 0.951)	0.972 (0.782, 0.991)	0.4089	*p* < 0.001
	RMSE (NM/kg)	0.029 (0.028~0.083)	0.021 (0.015~0.035)	0.096 (0.058~0.145)	0.033 (0.025~0.083)	0.3683	*p* < 0.001

*** *p* < 0.001.

**Table 6:** Model accuracy in muscle excitations.

		EMGAss	EMGAss_US	SO	SO_US	Effect Size	*p*-Value
Symptomatic Side							
R^2^	BFL	0.673 (0.6, 0.7)	0.811 (0.7, 0.9)	0.109 (0.1, 0.2)	0.239 (0.1, 0.3)	0.7149	*p* < 0.001
	RF	0.278 (0.1, 0.4)	0.260 (0.1, 0.5)	0.111 (0.1, 0.2)	0.152 (0.1, 0.3)	0.0897	0.050
	ST	0.630 (0.5, 0.7)	0.850 (0.8, 0.9)	0.083 (0.0, 0.2)	0.224 (0.1, 0.4)	0.7041	*p* < 0.001
	VL	0.284 (0.2, 0.3)	0.376 (0.2, 0.5)	0.068 (0.0, 0.1)	0.132 (0.1, 0.2)	0.4171	*p* < 0.001
	VM	0.312 (0.2, 0.5)	0.371 (0.3, 0.5)	0.074 (0.1, 0.1)	0.151 (0.1, 0.3)	0.461	*p* < 0.001
RMSE	BFL	0.084 (0.0, 0.1)	0.064 (0.0, 0.1)	0.081 (0.0, 0.2)	0.096 (0.1, 0.2)	0.0689	0.112
	RF	0.127 (0.1, 0.2)	0.075 (0.0, 0.1)	0.084 (0.1, 0.1)	0.120 (0.1, 0.2)	0.0785	0.077
	ST	0.062 (0.0, 0.1)	0.063 (0.0, 0.1)	0.075 (0.0, 0.1)	0.094 (0.0, 0.2)	0.0605	0.153
	VL	0.138 (0.1, 0.2)	0.079 (0.1, 0.1)	0.098 (0.1, 0.2)	0.117 (0.1, 0.2)	0.0808	0.071
	VM	0.102 (0.0, 0.1)	0.098 (0.1, 0.1)	0.091 (0.1, 0.2)	0.127 (0.1, 0.2)	0.0311	0.439
Asymptomatic Side							
R^2^	BFL	0.618 (0.4, 0.7)	0.754 (0.7, 0.8)	0.100 (0.1, 0.1)	0.131 (0.1, 0.2)	0.7966	*p* < 0.001
	RF	0.398 (0.3, 0.5)	0.439 (0.3, 0.5)	0.051 (0.0, 0.1)	0.119 (0.1, 0.2)	0.6326	*p* < 0.001
	ST	0.604 (0.5, 0.8)	0.769 (0.7, 0.9)	0.079 (0.0, 0.1)	0.147 (0.1, 0.3)	0.7736	*p* < 0.001
	VL	0.284 (0.2, 0.5)	0.550 (0.3, 0.6)	0.042 (0.0, 0.1)	0.069 (0.1, 0.2)	0.6645	*p* < 0.001
	VM	0.272 (0.2, 0.5)	0.414 (0.2, 0.6)	0.071 (0.0, 0.1)	0.062 (0.0, 0.1)	0.5334	*p* < 0.001
RMSE	BFL	0.099 (0.1, 0.1)	0.052 (0.0, 0.1)	0.151 (0.1, 0.2)	0.159 (0.1, 0.2)	0.239	*p* < 0.001
	RF	0.114 (0.1, 0.2)	0.072 (0.0, 0.1)	0.182 (0.1, 0.2)	0.161 (0.1, 0.2)	0.3973	*p* < 0.001
	ST	0.088 (0.1, 0.1)	0.049 (0.0, 0.1)	0.157 (0.1, 0.2)	0.170 (0.1, 0.2)	0.3106	*p* < 0.001
	VL	0.129 (0.1, 0.2)	0.105 (0.1, 0.1)	0.213 (0.1, 0.3)	0.166 (0.1, 0.3)	0.2188	*p* < 0.001
	VM	0.107 (0.1, 0.1)	0.094 (0.1, 0.1)	0.181 (0.1, 0.3)	0.177 (0.1, 0.2)	0.2079	*p* < 0.001
Healthy Group							
R^2^	BFL	0.610 (0.4, 0.8)	0.740 (0.7, 0.9)	0.089 (0.0, 0.2)	0.263 (0.1, 0.3)	0.6896	*p* < 0.001
	RF	0.364 (0.1, 0.5)	0.561 (0.3, 0.6)	0.053 (0.0, 0.1)	0.068 (0.0, 0.2)	0.4151	*p* < 0.001
	ST	0.530 (0.4, 0.7)	0.807 (0.7, 0.9)	0.123 (0.1, 0.3)	0.227 (0.1, 0.4)	0.6037	*p* < 0.001
	VL	0.344 (0.3, 0.4)	0.542 (0.3, 0.6)	0.060 (0.0, 0.1)	0.091 (0.0, 0.3)	0.4784	*p* < 0.001
	VM	0.300 (0.2, 0.5)	0.531 (0.4, 0.6)	0.063 (0.0, 0.1)	0.093 (0.0, 0.2)	0.6628	*p* < 0.001
RMSE	BFL	0.072 (0.0, 0.1)	0.050 (0.0, 0.1)	0.171 (0.1, 0.3)	0.086 (0.1, 0.2)	0.3217	*p* < 0.001
	RF	0.078 (0.1, 0.2)	0.061 (0.0, 0.1)	0.208 (0.1, 0.2)	0.126 (0.1, 0.2)	0.2282	*p* < 0.001
	ST	0.049 (0.0, 0.1)	0.048 (0.0, 0.1)	0.197 (0.1, 0.3)	0.070 (0.0, 0.2)	0.3234	*p* < 0.001
	VL	0.061 (0.0, 0.1)	0.051 (0.0, 0.1)	0.177 (0.1, 0.2)	0.105 (0.1, 0.1)	0.413	*p* < 0.001
	VM	0.080 (0.1, 0.2)	0.064 (0.0, 0.1)	0.179 (0.2, 0.3)	0.113 (0.1, 0.2)	0.4474	*p* < 0.001

**Table 7:** Comparisons of muscle force dispariti es.

		KOA Group		Healthy Group	Effect Size	*p*-Value
		Symptomatic Side	Asymptomatic Side			
Average Force						
	RF	78.418 (64.2, 94.0)	71.479 (52.3, 194.4)	112.912 (72.5, 306.4)	1.521	*p* < 0.001
	VL	175.765 (129.6, 265.1)	173.661 (95.6, 226.6)	90.784 (58.8, 133.2)	2.2431	*p* < 0.001
	VM	142.664 (98.1, 174.1)	94.909 (56.5, 141.5)	54.273 (22.9, 81.0)	3.825	*p* < 0.001
	BFL	144.351 (54.3, 316.0)	114.009 (27.2, 213.5)	119.902 (46.8, 258.9)	5.1531	*p* < 0.001
	ST	24.324 (17.5, 36.1)	59.768 (38.8, 94.1)	26.126 (12.4, 49.1)	6.6557	*p* < 0.001
Peak Force						
	RF	100.983 (65.3, 110.9)	94.840 (64.9, 384.9)	242.126 (122.6, 484.7)	3.9715	0.006 **
	VL	374.857 (213.2, 470.0)	264.927 (209.6, 302.5)	150.715 (103.5, 218.7)	5.6596	0.004 **
	VM	197.398 (172.9, 214.9)	174.324 (117.4, 196.0)	96.089 (52.5, 137.3)	6.4636	0.001 **
	BFL	507.825 (83.0, 780.3)	365.836 (33.2, 402.7)	226.675 (140.3, 721.6)	0.0889	0.463
	ST	53.530 (44.6, 61.0)	104.713 (96.3, 189.8)	65.384 (29.7, 140.1)	1.8047	0.003 **

** *p* < 0.01.

**Table 8:** Comparisons of knee contact force disparity.

		KOA Group		Healthy Group	Effect Size (|r|)	*p*-Value
		Symptomatic Side	Asymptomatic Side			
Average Force						
	LCF	439.600 (212.9,668.7)	373.825 (234.8, 639.7)	646.955 (273.5, 1007.8)	0.6375	0.003 *
	MCF	1681.998 (1470.9, 1960.4)	1629.830 (1348.5, 2004.6)	1203.959 (922.3, 2025.8)	0.0842	*p* < 0.001
	Total	2130.701 (1747.9, 2565.6)	2000.975 (1583.2, 2637.7)	1706.516 (1435.6, 2711.1)	0.2384	*p* < 0.001
Peak Force						
	LCF 1st	478.610 (382.0, 610.7)	559.000 (419.5, 710.8)	700.500 (511.2, 733.6)	2.6028	0.010 *
	MCF 1st	1860.490 (1707.7, 2311.8)	1791.950 (1559.5, 1930.5)	1574.070 (1389.0, 1852.3)	3.0567	0.011 *
	Total 1st	2537.925 (2186.8, 2706.1)	2369.027 (2214.8, 2615.0)	2129.655 (1849.0, 2384.5)	0.7539	0.010 *
	LCF 2st	365.425 (237.5, 453.4)	434.000 (278.8, 616.5)	609.500 (505.5, 699.6)	2.9172	0.002 **
	MCF 2st	1645.450 (1416.2, 1884.4)	1582.050 (1301.8, 1773.3)	1491.500 (1219.3, 1714.0)	2.4819	0.312
	Total 2st	2261.240 (2169.6, 2566.1)	2035.000 (1756.3, 2482.2)	2037.165 (1625.5, 2633.3)	1.6063	0.320

* *p* < 0.05 ** *p* < 0.01. LCF Lateral knee contact force, MCF Medial knee contact force.

## Data Availability

The datasets in the current study are available from the corresponding author on reasonable request.
